# Ciclosporin A Proof of Concept Study in Patients with Active, Progressive HTLV-1 Associated Myelopathy/Tropical Spastic Paraparesis

**DOI:** 10.1371/journal.pntd.0001675

**Published:** 2012-06-12

**Authors:** Fabiola Martin, Hannah Castro, Carolyn Gabriel, Adine Adonis, Alexandra Fedina, Linda Harrison, Liz Brodnicki, Maria A. Demontis, Abdel G. Babiker, Jonathan N. Weber, Charles R. M. Bangham, Graham P. Taylor

**Affiliations:** 1 Centre for Immunology and Infection, Department of Biology, Hull and York Medical School, University of York, York, United Kingdom; 2 Medical Research Council, Clinical Trials Unit, London, United Kingdom; 3 Department of Neurology, St Mary's Hospital, London, United Kingdom; 4 National Centre for Human Retrovirology, St Mary's Hospital, London, United Kingdom; 5 Section of Infectious Diseases, Faculty of Medicine, Imperial College, London, United Kingdom; George Mason University, United States of America

## Abstract

**Introduction:**

Patients with HTLV-1-associated myelopathy/tropical spastic paraparesis (HAM/TSP) become progressively impaired, with chronic pain, immobility and bladder, bowel and sexual dysfunction. Tested antiretroviral therapies have not been effective and most patients are offered a short course of corticosteroids or interferon-α, physiotherapy and symptomatic management. Pathogenesis studies implicate activated T-lymphocytes and cytokines in tissue damage. We therefore tested the hypothesis that inhibition of T-cell activation with ciclosporin A would be safe and clinically beneficial in patients with early and/or clinically progressing HAM/TSP.

**Materials and Methods:**

Open label, proof of concept, pilot study of 48 weeks therapy with the calcineurin antagonist, ciclosporin A (CsA), in seven patients with ‘early’ (<two years) or ‘progressive’ (>50% deterioration in timed walk during the preceding three months) HAM/TSP. Primary outcomes were incidence of clinical failure at 48 weeks and time to clinical failure.

**Results:**

All patients completed 72 weeks study participation and five showed objective evidence of clinical improvement after 3 months treatment with CsA. Two patients exhibited clinical failure over 6.4 person-years of follow-up to week 48. One patient had a >2 point deterioration in IPEC (Insituto de Pesquisa Clinica Evandro Chagas) disability score at weeks 8 and 12, and then stopped treatment. The other stopped treatment at week 4 because of headache and tremor and deterioration in timed walk, which occurred at week 45. Overall pain, mobility, spasticity and bladder function improved by 48 weeks. Two patients recommenced CsA during follow-up due to relapse.

**Conclusions:**

These data provide initial evidence that treatment with CsA is safe and may partially reverse the clinical deterioration seen in patients with early/progressive HAM/TSP. This trial supports further investigation of this agent's safety and effectiveness in larger, randomised controlled studies in carefully selected patients with disease progression.

## Author Summary

HTLV-1 is a retrovirus transmitted through body fluids that is commonly seen in the West Indies, South America and Southern Japan but rarely in the UK. Although most patients remain healthy carriers, HTLV-1 causes serious conditions such as adult T cell leukaemia/lymphoma (ATLL) and HTLV-1-associated myelopathy/Tropical Spastic Paraparesis (HAM/TSP). The infection which is life-long cannot be eradicated and treatments for the associated diseases are limited. We report the encouraging findings of the first UK Medical Research Council funded treatment study for patients with early and/or deteriorating HAM/TSP. Treatment with ciclosporin A, a drug commonly used to dampen the immune system in transplant patients, was investigated. Symptoms and signs of disease, particularly low back pain and muscle stiffness, improved by week 24 and in some patients this improvement persisted after the 48 weeks of treatment, at least to the end of the study at week 72. Most striking was the finding that the amount of HTLV-1 in the fluid around the spinal cord, called cerebrospinal fluid, was reduced during treatment. These findings justify the further study of ciclosporin A in patients with HAM/TSP.

## Introduction

An estimated 20 million people are infected with HTLV-1 worldwide [1] of which approximately 8% (∼2 million) will develop HTLV-1-associated diseases, most notably adult T-cell leukaemia/lymphoma (ATLL) [2], HTLV-1-associated myelopathy/tropical spastic paraparesis (HAM/TSP) [3], HTLV-1-associated uveitis (HAU) [4] and infective dermatitis [5].

An association between myelopathy and HTLV-1 seropositivity in serum and cerebrospinal fluid (CSF) was first reported in 1985, in patients with Tropical Spastic Paraparesis (TSP) in Martinique [3], and independently in Japan in 1986, where the condition was termed HTLV-1-associated myelopathy (HAM) [6]. It is accepted that HAM/TSP, a chronic and progressive spastic paraparesis in patients living in both endemic and non-endemic countries [7], [8], [9], is an inflammatory condition [10].

But almost thirty years after its discovery little is known about how best to treat HAM/TSP [11]. Although corticosteroids [12], [13] and interferon-α/β [14] have been used as therapy for HAM/TSP, they are only of transient benefit (weeks), cause serious side-effects and have not been compared to placebo.

Particular challenges to designing clinical trials in HAM/TSP include the uncertainties relating to the following questions:

- Does the stage of disease influence treatment outcome?
- Which treatment approach should be adopted – antiviral or anti-inflammatory?
- How long should treatment be given?

In 2006 a revision of the original WHO diagnostic criteria of HAM/TSP allowed ascertainment of three diagnostic levels: definite, probable and possible after excluding all conditions that could mimic HAM/TSP [15]. To monitor clinical progression of HAM/TSP and therapy response, clinicians now differentiate between i) early and late ii) clinically progressive and non-progressive and iii) active and inactive definite HAM/TSP [11],[16] (and personal communication with the HAM/TSP clinical trial subgroup). Immunosuppressive therapy is especially attractive in patients with early, clinically progressive, or active HAM/TSP, which are defined as onset of symptoms ≤2 years, evidence of clinical progression, such as deterioration of timed walk or progression in disability scales, or evidence of CNS inflammatory activity from cerebrospinal fluid (CSF) analysis retrospectively.

However immunosuppressive therapy given to patients with HTLV-1 infection may increase the chance of developing ATLL. Cases of ATLL following organ transplantation, for which much greater immunosuppression is prescribed than in this study, have been reported but the risk has not been quantified for any specific immunosuppressive treatment [17], [18], [19], [20], [21], [22].

Also the optimal duration of any therapy for patients with HAM/TSP has never been examined and due to its side-effects long-term administration of corticosteroids, the most commonly prescribed first line treatment, is not recommended.

With the aim to identify a long-term, safe corticosteroid-sparing immunosuppressive therapy for patients with HAM/TSP, we conducted an open, proof of principle study, which examined the potential clinical and immunological efficacy and the safety of Ciclosporin A (CsA) in patients with early, progressive HAM/TSP (September 2006 to January 2010, NCT00773292).

CsA is used routinely and long-term in post-allergenic organ transplants as well as inflammatory dermatological and musculoskeletal disease such as psoriasis, ectopic dermatitis and rheumatoid arthritis. It has also been used for idiopathic uveitis and severe cases of immune-mediated haemolytic anaemia.

The primary objective of this study was to determine whether CsA treatment for 48 weeks improved clinical outcome measures of patients with early or progressing definite HAM/TSP. The secondary objectives were to determine i) the safety of CsA, ii) the effects of CsA on HTLV-1 proviral load in blood and CSF and iii) the effect of CsA on markers of T-cell activation and proliferation. The study also explored the mechanism of pathogenesis of HAM/TSP by analysing the effect of CsA on the severity and progression of HAM/TSP.

## Materials and Methods

### Study design

The study protocol, patient information sheet and consent form were submitted to the National Research Ethics Service and approved by the Oxfordshire ‘A’ Research Ethics Committee (Reference 06/Q1604/75); Clinical Trial Registration: Clinicaltrials.gov: NCT00773292. Eligible patients had definite HAM/TSP, as defined by ‘Belem criteria’ [15] and either had developed first symptoms within the last two years or had progressive disease, as defined by ≥50% documented deterioration in 10 m timed walk over the preceding three months. All patients were older than 16 years, not pregnant or breastfeeding, and were serologically negative when tested for HIV 1/2, HBV, HCV, syphilis and *Strongyloides stercoralis*. There was no evidence of pulmonary tuberculosis on chest x-rays and none of the patients had a history of malignant or autoimmune disease. After giving written informed consent, patients attending the National Centre for Human Retrovirology at St Mary's Hospital, London, underwent two baseline visits at least one day and no more than four weeks apart and trial therapy was commenced after the 2^nd^ baseline visit. Patients were recruited sequentially and initially treated with oral CsA 2.5–5 mg/kg/day divided into two, 12-hourly doses and subsequently dose-adjusted to maintain plasma trough levels between 80–100ng/ml for 48 weeks with CsA plasma concentrations monitored at each on-treatment study visit. Post-treatment patients were followed for an additional 24 weeks.

The primary outcome measures were i) incidence of clinical failure at 48 weeks and ii) time to clinical failure. Clinical failure was defined as any of the following i) lack of any objective improvement after three months of therapy, ii) greater than two point deterioration (increase) in the IPEC 1 scale compared with baseline at two consecutive visits excluding weeks 2 and 4 and iii) ≥30% deterioration in timed walk compared with baseline at any time during the trial. Objective improvement was defined as any of the following comparing baseline measurements to 12, 24 and 48 weeks: i) one point decrease in the IPEC 1 scale (Instituto de Pesquisa Clínica Evandro Chagas), ii) >30% improvement in 10 m timed walk, iii) visual analogue pain score reduced by >2 points, iv) reduction of frequency or nocturia by greater than one or reduction of residual volume by more than 10% at two consecutive visits.

Secondary outcomes were the following clinical and laboratory variables measured at 12, 24, 48 and 72 weeks: Seconds needed to walk 10 meters (sec/10 m TW), change in walking aid, maximum pain (visual analogue score 0–10/10 cm VAS), modified Ashworth scale score (MASS, Appendix S1), walking scale (MSWS-12, Appendix S2), IPEC 1 and 2 disability scale score (Appendix S3), bladder sphincter function (daily self-assessment and ultrasonic measurement of the residual and urgency urinary volume), spasticity scale (SPAST-88, Appendix S4), SF-36 quality of life scale (Appendix S5), HTLV-1 proviral load in venous blood and in CSF, total CD4+ lymphocytes, CD8+ lymphocytes and %CD4+CD25+ T lymphocytes counts and plasma β_2_ microglobin as inflammatory markers.

#### Statistical methods

Data were collected at baseline (2 visits), week 2, week 4 and then four weekly until week 24, 8-weekly until week 48 and 12-weekly to week 72. Data were entered into Oracle databases, and checked manually and by computer consistency checks. Analysis text files were created from the database and imported into STATA (version 11; Stata Corporation, College Station, Texas, USA) for statistical analysis. Week 0 was defined as the date of the second baseline visit; for numerical variables, the baseline measurement was defined as the average of the measurements taken at screening (first baseline visit) and week 0 (second baseline visit). Intention to treat (ITT), data from all time-points on all patients who commenced CsA even if they subsequently stopped the drug, and per-protocol (PP) analysis, data from time-points up to discontinuation of CsA or re-start of CsA after 48 weeks for secondary outcomes, were performed. Secondary outcomes were analysed as average change from baseline to a given time point as measured by area under the curve minus baseline (AUCMB) using the t-test. Comparisons were also made between the AUCMB at 24 weeks with the AUC minus week 24 (AUCM24) at 48 weeks, and the AUCMB at 48 weeks with the AUC minus week 48 (AUCM48) at 72 weeks, using paired t-tests. Missing values at baseline, week 24 or week 48, in order to calculate AUCMB, AUBM24 and AUCM48 were approximated by linear extrapolation to the two nearest values. As CSF outcomes were only measured at week 12, changes from baseline for these outcomes were analysed by regression analysis. Timed walk rank was calculated by ranking the time to walk 10 meters over all patients and visits, in the following order (highest/worst score to lowest/best score): unable to walk; able to walk, but not able to complete 10 meters (ranked on distance walked and time taken); able to walk 10 meters with a bilateral aid; able to walk 10 meters with a unilateral aid; able to walk 10 meters unaided (all ranked on time taken). For example, there were 99 occasions when timed walk was recorded for all patients; patient HC036005 was the fastest patient on these 99 occasions to walk 10 m, unaided, taking 7 seconds at each week 2, 4, 8, 12 and 24 visits, so this patient at these visits was given the lowest score of 1; patient HC036006 did not complete a timed walk at 0 and 2 week visits since wheelchair bound, so this patient at these visits was given the highest score of 98; all patients and visits in-between these extremes were ranked in order as described above.tpb

## Results

### Study population, treatment and follow up

**Figure 1:**
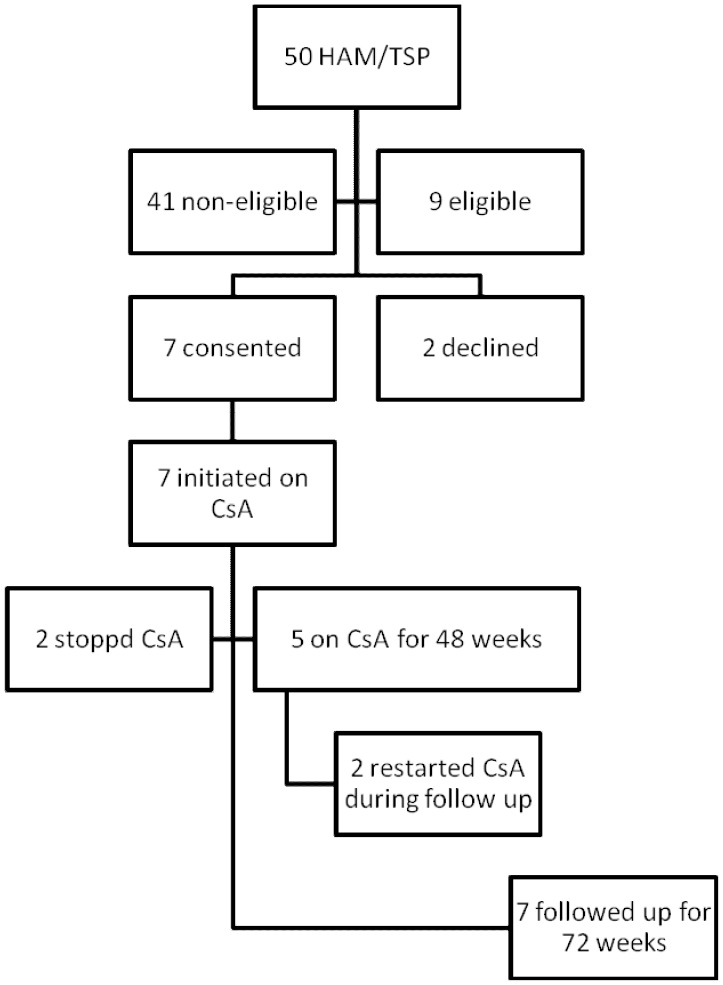
Flowchart of enrolment and follow up of patients with definite and early and/or progressing HAM/TSP. 50 patients with HAM/TSP were identified and 9 were identified as patients with early and/or deteriorating HAM/TSP, 7 of whom consented to treatment with ciclosporin A. Two patients chose to re-start ciclosporin A after 48 weeks during trial period.

**Figure 2:**
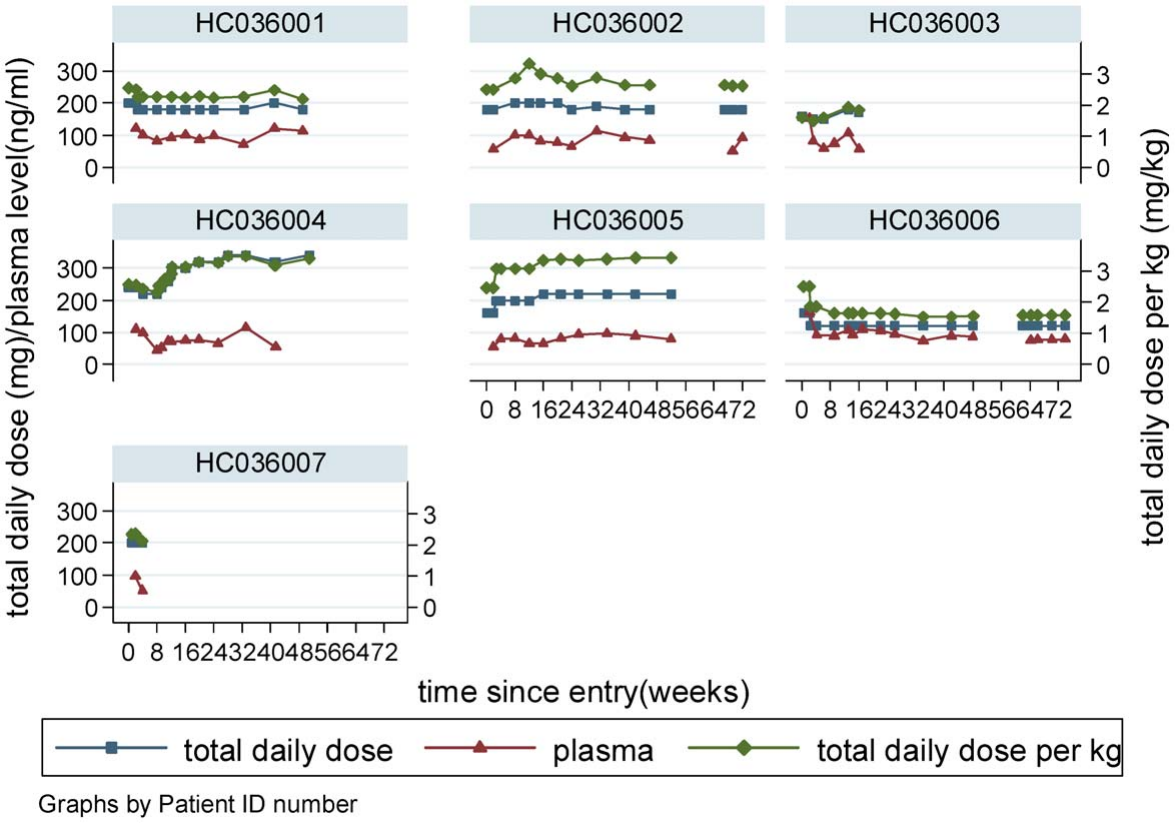
Therapeutic drug monitoring of ciclosporin A: dosing and through CsA plasma concentrations.

We aimed to recruit eight patients. Of the nine eligible patients with definite, early or progressing HAM/TSP informed of the study, seven agreed to participate (Figure 1). Baseline demographic, clinical and laboratory characteristics of the patients are shown in Table 1. Patients were on average 50 years old (range 40–69), mostly female (5 of 7) and of Afro-Caribbean origin having acquired HTLV-1 either through mother-to-child transmission or sexual intercourse. As per protocol one patient with early disease (0.8 years) was entered into the study without a three months follow up period. The remainder had progressing disease with documented deterioration in timed walk >50% over the previous three months. All seven patients initiated treatment with body weight adjusted CsA (2.5 mg/kg/day) with a median daily dose of 180 mg (range 160–240). The median trough CsA concentration from the 59 measurements was 87 mg/ml (range 46–164) with 44% of all measurements within the target range (Figure 2). Patients were followed up for a median of 74 weeks (range 71–92).

**Table 1:** Demographics, and baseline clinical and laboratory measures of patients with HAM/TSP receiving ciclosporin A (n = 7); median (ranges) or number (%) given.

Age (years)	50 (40–69)
Female	5 (71%)
Likely mode of infection:	
Mother to child	3 (43%)
Sexual intercourse	1 (14%)
Mother to child or sexual intercourse	3 (43%)
Ethnic origin:	
Afro-Caribbean	5 (71%)
White	2 (28%)
Body weight (kg)	79 (64–100)
Duration of HAM/TSP (years)	4.6 (0.8–17.3)
**Clinical measures – objective**	
Time to walk (seconds/10 meter)	29.5 (8.0–68.0)
1 patient unaided	8
2 patients unilateral walking aid	27.3 (25.0–29.5)
4 patients bilateral walking aid	50.3 (27.0–68.0)
IPEC 1 Disability Scale Score (min 0- max 29)	14.5 (2.5–18.5)
Modified Ashworth Scale Score (min 0- max 4)	1.8 (0.0–2.5)
Bladder Residual volume^a^ (ml)	8 (0–267)
Bladder Urge volume^a^ (ml)	237 (190–417)
**Clinical measures – subjective**	
Maximum pain (min 0- max 10)	8.4 (0.0–9.5)
IPEC 2 Disability Scale Score (min 0- max 43.25)	32.7 (15.5–40.5)
Daily urinary frequency^a^ (per day)	5 (4–7)
Nocturia frequency^a^ (per night)	3 (1–5)
Number of episodes of incontinence^a^ (per 24 hours)	0.0 (0–0.2)
Total MSWS-12 (transformed^b^)	93 (19–96)
Average MSWS-12 (min 1- max 5)	4.7 (1.8–4.8)
Total Spasticity Scale-88 score (transformed^b^)	591 (113–736)
Total SF-36 Score (transformed^b^)	218 (100–610)
**Laboratory measures**	
HTLV-1 proviral DNA (blood) (copies/100 PBMCs)	14.4 (4.8–32.0)
Log_10_ HTLV-1 proviral DNA (blood) (copies/100 PBMCs)	1.2 (0.7–1.5)
Protein (CSF) (g/L)	0.46 (0.34–0.62)
WCC (CSF) (10^6^/L)^c^	3.5 (1–8)
Log_10_ HTLV-1 proviral DNA (CSF) (copies/100 nucleated cells)	1.7 (1.5–2.6)
CSF/PBMC ratio	4.4 (1.0–17.1)
CD4 absolute cell count (10^6^/L)	1595 (805–2260)
CD4%	53 (47–62)
CD8 absolute cell count (10^6^/L)	690 (230–1195)
CD8%	29 (15–35)
CD25%	43 (37–62)
CD4/CD25%	14 (7–30)
CD4/CD25 absolute (10^6^/L)	203 (94–526)
Total lymphocytes (10^6^/L)	3000 (1350–3500)
Absolute HTLV-1 proviral burden (total lymphocytes 10^6^/L×copies/100PBMCs)	26369 (6461–111983)
β-2 microgobulin (mg/L)	2.6 (1.6–6.1)

a : N = 5, 2 patients had catheters;

b : (observed score-min possible score)/(max possible score-min possible score)×100;

c : N = 6. Abbreviations: IPEC disability scale = Insituto de Pesquisa Clinica Evandro Chagas; MSWS = multiple sclerosis walking scale; WCC = white cell count; CSF = cerebrospinal fluid; PBMC = peripheral blood mononuclear cells.

### Primary outcomes

Five patients (72%) completed the 48 weeks of treatment without interruption. Two of these patients requested to re-start CsA during the 24 week post therapy follow-up period due to clinical deterioration which they attributed to stopping CsA.

Over 6.4 person-years follow-up to 48 weeks, no patient failed according to the first primary outcome criterion i.e. objective improvement was observed in all patients.

However four patients met the 2^nd^ and 3^rd^ criteria for clinical failure by 48 weeks, which gives an incidence of 0.6 per person-year, although only two were true clinical failures as detailed below.

One patient failed on the second criterion with a >2 point deterioration of IPEC1 scale on two consecutive visits during therapy at visit weeks 8 and 12. This patient, with a history of urinary tract infections (UTI), developed a further UTI and became depressed during treatment with CsA, which was discontinued at week 16. Of the three patients who failed due to >30% deterioration in TW (3^rd^ criterion), two were technical failures (at weeks 4 and 33 respectively) with temporary deteriorations due to accidents rather than progression of HAM/TSP and both continued CsA. The third patient developed headache and tremor attributed to CsA which was discontinued at week four but the deterioration in TW was documented at week 45, off treatment. By intention-to-treat, 2/7 patients clinically failed this trial, and both had to discontinue CsA due to side-effects.

### Secondary outcomes (Table 2)

#### Objective clinical measures

**Figure 3:**
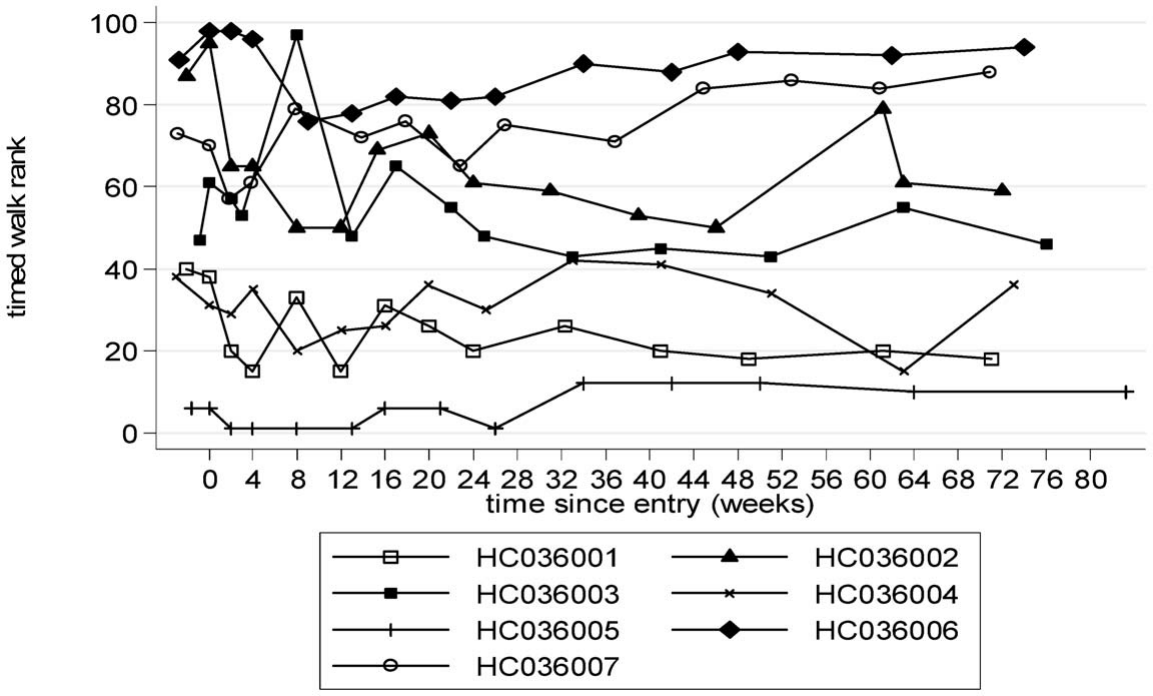
Timed walk ranked by walking aid used (0–72 weeks). A higher rank represents a slower walk. HC036005 walked unaided; HC036001, HC036004 one walking stick; HC036002, HC036003, HC036006, HC036007 needed 2 walking sticks. At week 0 and 2 HC036006 needed a wheelchair due to a sprained ankle.

**Figure 4:**
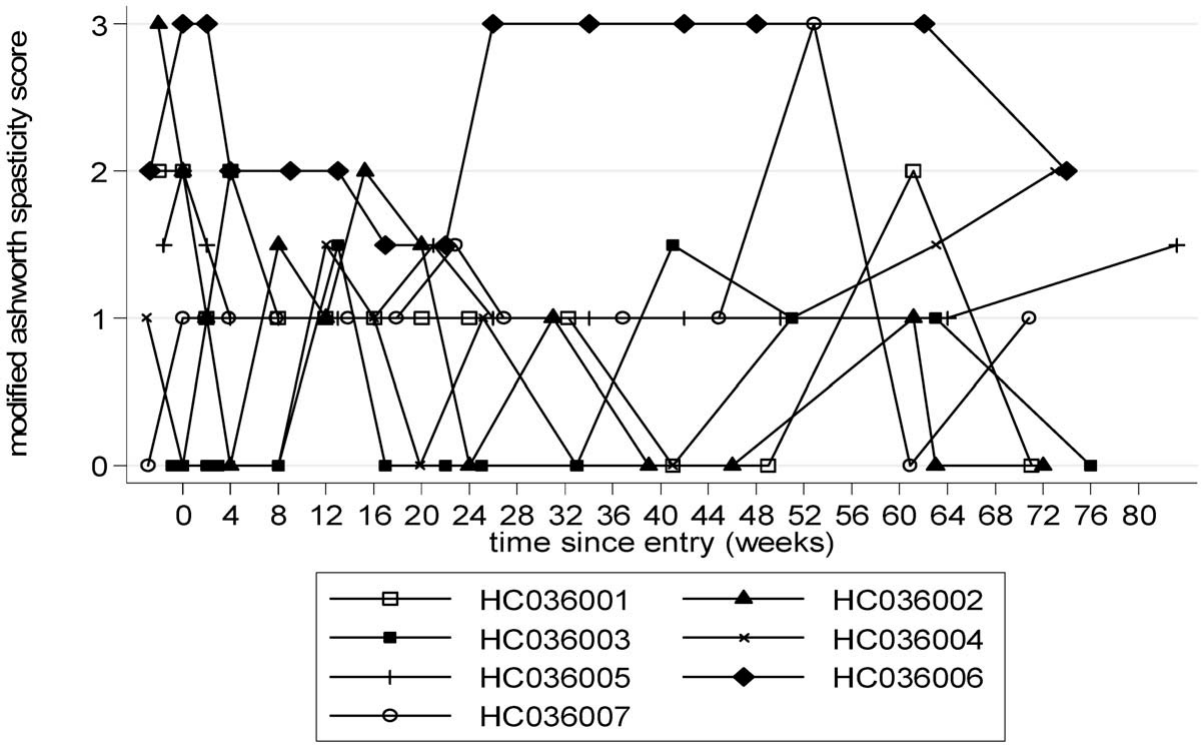
Modified Ashworth Scale testing muscle spasticity (0–72 weeks). A higher score represents increased muscle tone.

Trends in timed walk were biased by the two patients whose accidents during the study caused a marked, but reversible, deterioration in timed walk. Therefore timed walk rank, which takes into account walking aids, was analysed. This showed a trend towards improvement compared to baseline at 12 weeks with a mean (SE) decrease in score of −7 (5), p = 0.24 (ITT, Table 2), which was maintained to week 48 and to week 72 (after treatment had stopped), by ITT and PP analysis (Figure 3). Compared to baseline changes in IPEC-1 were small with or without treatment (Table 2). Spasticity (MASS) improved at all time points compared to baseline, with a decrease in score of −0.3 or −0.4, although changes did not reach statistical significance (Figure 4, Table 2). Ultrasonic measurements of residual and urge volume were difficult to interpret because two patients had urinary catheters.

**Table 2:** Secondary outcomes measured in patients with HAM/TSP receiving Ciclosporin A.

Intention to treat analysis (N = 7)	Area under the curve minus…	mean (SE)	p-value	p-value
**Time to walk rank**	baseline to 12 weeks	−7 (5)	0.24	-
	baseline to 24 weeks	−8 (4)	0.14	-
	baseline to 48 weeks	−8 (5)	0.14	-
	24 weeks to 48 weeks	3 (2)	0.28	0.07^a^
	48 weeks to 72 weeks	1 (2)	0.75	0.22^b^
**Modified Ashworth Scale Score**	baseline to 12 weeks	−0.3 (0.2)	0.2	-
	baseline to 24 weeks	−0.3 (0.3)	0.23	-
	baseline to 48 weeks	−0.4 (0.3)	0.29	-
	24 weeks to 48 weeks	0.0 (0.2)	0.86	0.31^a^
	48 weeks to 72 weeks	0.1 (0.2)	0.54	0.32^b^
**Maximum pain**	baseline to 12 weeks	−0.9 (0.4)	0.04	-
(visual analogue scale)	baseline to 24 weeks	−1.2 (0.5)	0.05	-
	baseline to 48 weeks	−1.6 (0.8)	0.09	-
	24 weeks to 48 weeks	−0.1 (0.5)	0.78	0.13^a^
	48 weeks to 72 weeks	0.8 (0.7)	0.27	0.09^b^
**IPEC 1 Disability Scale Score**	baseline to 12 weeks	−0.04 (0.90)	0.97	
	baseline to 24 weeks	0.03 (0.85)	0.97	
	baseline to 48 weeks	0.03 (0.79)	0.98	
	24 weeks to 48 weeks	−0.05 (0.31)	0.87	0.92^a^
	48 weeks to 72 weeks	0.78 (0.38)	0.08	0.30^b^
**IPEC 2 Disability Scale Score**	baseline to 12 weeks	−2.0 (2.1)	0.38	
	baseline to 24 weeks	−2.5 (2.2)	0.31	
	baseline to 48 weeks	−1.2 (1.8)	0.54	
	24 weeks to 48 weeks	4.1 (1.8)	0.06	0.08^a^
	48 weeks to 72 weeks	0.1 (0.8)	0.88	0.43^b^
**Daily urinary frequency** c	baseline to 12 weeks	−0.8 (0.2)	0.02	
	baseline to 24 weeks	−0.9 (0.2)	0.01	
	baseline to 48 weeks	−0.9 (0.2)	0.01	
	24 weeks to 48 weeks	−0.1 (0.2)	0.7	0.08^a^
	48 weeks to 72 weeks	−0.3 (0.1)	0.08	0.08^b^
**Nocturia frequency** c	baseline to 12 weeks	−1.0 (0.5)	0.13	
	baseline to 24 weeks	−1.2 (0.5)	0.08	
	baseline to 48 weeks	−1.3 (0.6)	0.1	
	24 weeks to 48 weeks	−0.2 (0.2)	0.47	0.07^a^
	48 weeks to 72 weeks	0.5 (0.3)	0.11	0.06^b^
**log_10_ HTLV-1 DNA** (blood)	baseline to 12 weeks	−0.12 (0.07)	0.16	-
(copies/100 PBMCs)	baseline to 24 weeks	−0.11 (0.08)	0.2	-
	baseline to 48 weeks	−0.12 (0.06)	0.08	-
	24 weeks to 48 weeks	−0.04 (0.07)	0.61	0.63^a^
	48 weeks to 72 weeks	0.05 (0.06)	0.4	0.12^b^
**Protein (CSF)** (g/L)	change from baseline to 12 wks^d^	0.13 (0.10)	0.24	-
**WCC (CSF)** (10^6^/L)^e^	change from baseline to 12 wks^d^	7.2 (3.0)	0.06	-
**log_10_ HTLV-1 DNA** (CSF)	baseline to 12 weeks	−0.19 (0.08)	0.05	-
	change from baseline to 12 wks^d^	−0.39 (0.15)	0.05	
**CSF/PBMC ratio**	baseline to 12 weeks	−2.2 (1.3)	0.14	-
	change from baseline to 12 wks^d^	−4.4 (2.6)	0.14	
**CD4 absolute cell count** (10^6^/L)	baseline to 12 weeks	−125 (84)	0.19	-
	baseline to 24 weeks	−231 (89)	0.04	-
	baseline to 48 weeks	−286 (94)	0.02	-
	24 weeks to 48 weeks	42 (28)	0.18	0.05^a^
	48 weeks to 72 weeks	64 (55)	0.29	0.03^b^
**CD8 absolute cell count** (10^6^/L)	baseline to 12 weeks	−60 (65)	0.39	-
	baseline to 24 weeks	−115 (70)	0.15	-
	baseline to 48 weeks	−127 (74)	0.14	-
	24 weeks to 48 weeks	62 (28)	0.06	0.08^a^
	48 weeks to 72 weeks	17 (39)	0.68	0.21^b^
**CD4/CD25%**	baseline to 12 weeks	−3.3 (2.6) −3.9 (2.7)	0.25	-
	baseline to 24 weeks	−4.4 (2.4)	0.2	-
	baseline to 48 weeks	−0.6 (1.3)	0.11	-
	24 weeks to 48 weeks	1.4 (1.5)	0.67	0.25^a^
	48 weeks to 72 weeks		0.39	0.07^b^
**β2microglobin** (mg/L)	baseline to 12 weeks	−0.09 (0.33)	0.77	-
	baseline to 24 weeks	−0.12 (0.39)	0.78	-
	baseline to 48 weeks	−0.31 (0.39)	0.45	-
	24 weeks to 48 weeks	−0.11 (0.14)	0.46	0.99^a^
	48 weeks to 72 weeks	0.03 (0.11)	0.77	0.50^b^

a : for difference between AUC minus baseline to 24 weeks and AUC minus 24 weeks at 48 weeks;

b : for difference between AUC minus baseline to 48 weeks and AUC minus 48 weeks at 72 weeks;

c : N = 5, 2 patients had catheters;

d : regression analysis;

e : N = 6. IPEC disability scale = Insituto de Pesquisa Clinica Evandro Chagas; WCC = white cell count; CSF = cerebrospinal fluid; PBMC = peripheral blood mononuclear cells.

#### Subjective clinical measures

**Figure 5:**
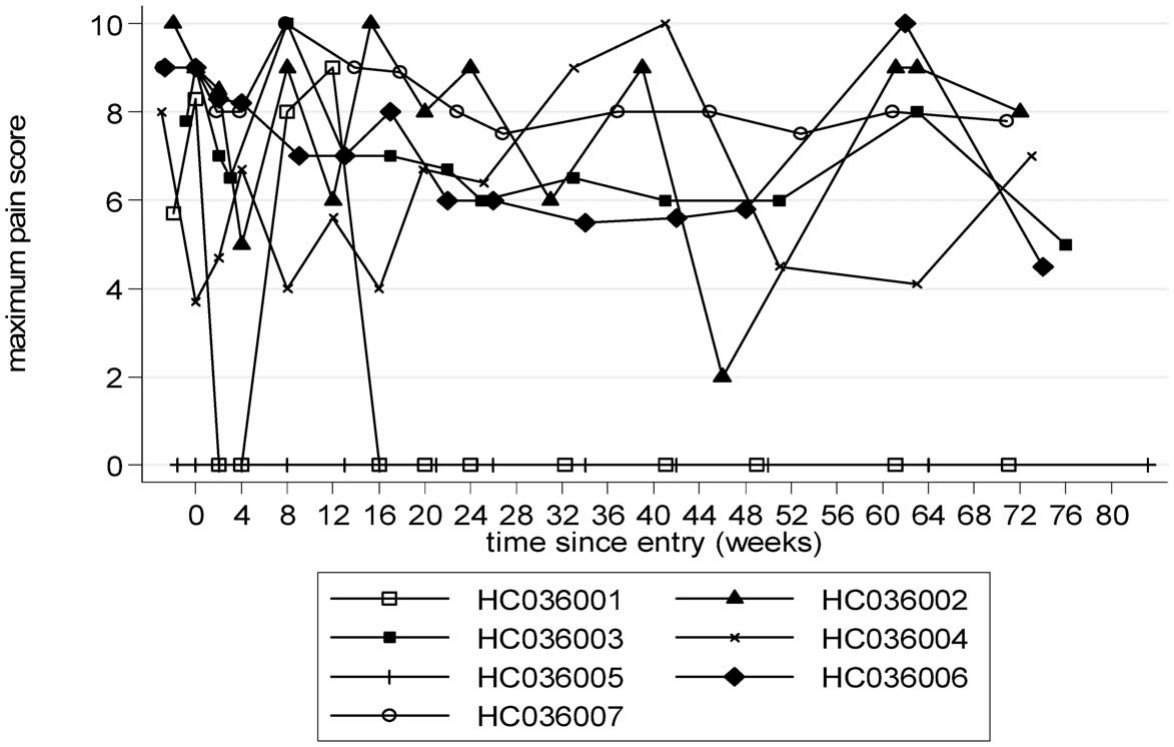
Maximum pain over time (0–72 weeks). A higher score represents more pain. If pain was reported at more than one site, the higher pain score was used.

The average maximum pain score (VAS) improved over time: (mean (SE) decrease compared to baseline at week 24 was ITT: 1.2 cm (0.5), p = 0.05; PP: 1.2 cm (0.5), p = 0.05) which was maintained to some degree post-treatment (Figure 5, Table 2). IPEC2 showed a trend towards improvement throughout follow up compared to baseline which was greatest in the first 24 weeks (mean (SE) decrease at week 24 and week 48 was ITT: −2.5 (2.2), p = 0.31 and −1.2 (1.8), p = 0.54 respectively; PP: −2.7 (2.0), p = 0.25 and −1.2 (1.8), p = 0.54 respectively; AUCM24 to 48 weeks p = 0.08 (ITT), 0.03 (PP)); the improvement continued to 72 weeks (Table 2). Urinary frequency improved with treatment (a mean decrease of approximately once a day/night), with nocturia improving slightly more than daytime frequency, although only daytime frequency changes reached statistical significance as there was more variation in nocturia frequencies, and this improvement was maintained to some degree post-treatment (Table 2). The number of episodes of incontinence did not change significantly either way during trial. Self-reported MSWS-12, SF-36 and SPAST-88 fluctuated over time but not markedly.

#### Laboratory measures

**Figure 6:**
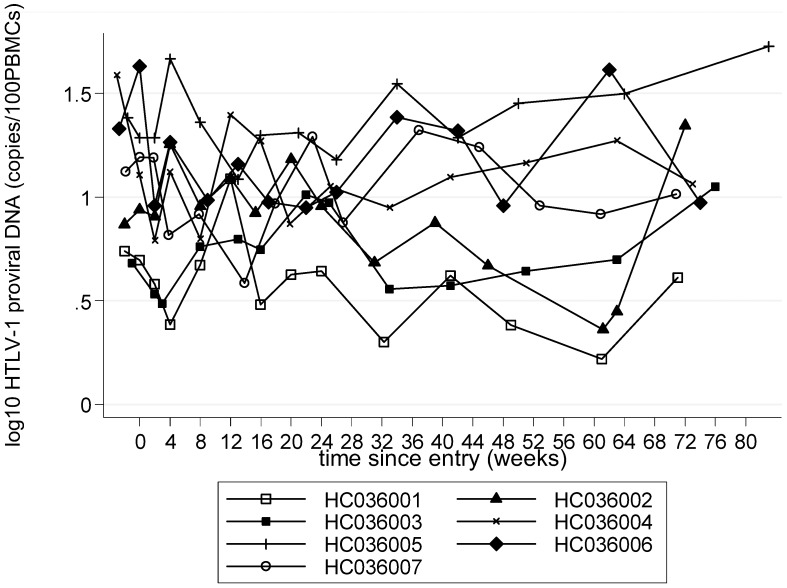
Log^10^ HTLV-1 proviral DNA in peripheral blood mononuclear cells (PBMCs) (0–72 weeks).

**Figure 7:**
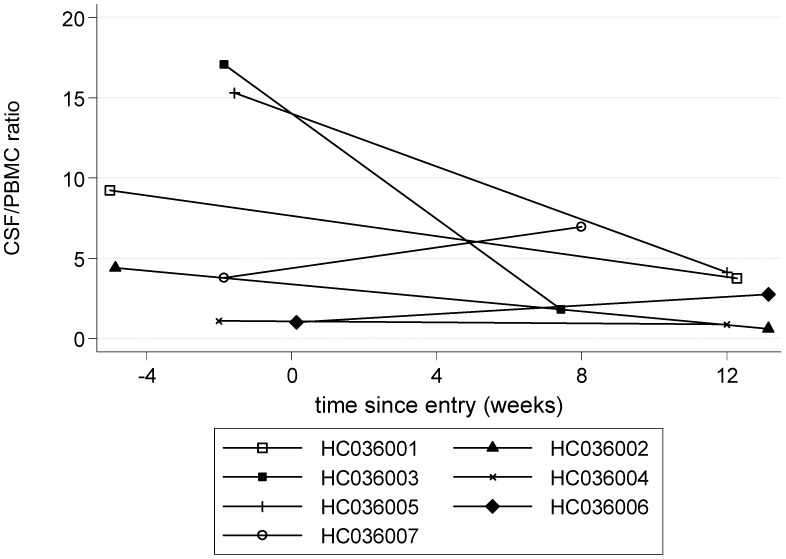
HTLV-1 proviral DNA of cerebro-spinal fluid over peripheral blood mononuclear cells (CSF/PBMC ratio) (0–12 weeks).

A small, mean (SE) 0.12 (0.06) log_10_, but consistent reduction in HTLV-1 proviral load in peripheral blood, measured as HTLV-1 DNA copies per 100 PBMCs, was observed during treatment, which persisted post-treatment (Figure 6, Table 2, Table 3). A mean (SE) 0.4 (0.15) log_10_ reduction in HTLV-1 proviral load in CSF at 12 weeks compared with baseline CSF examination was observed (p = 0.05, Table 2, 4). The ratio of CSF/blood viral load decreased more than four-fold during the first 12 weeks of CsA treatment (p = 0.14, Figure 7, Table 2) where as CSF WCC and protein increased by (mean (SE) 7.2 10^6^/L (3.0), p = 0.06, and 0.13 g/L (0.10), p = 0.24, compared to baseline, respectively).

**Table 3:** Log_10_ HTLV-1 proviral DNA (blood) (copies/100 PBMCs): 0–48 weeks whilst on CsA treatment; 49–72 weeks without treatment.

	Time since entry (weeks)													
	−2	0	2	4	8	12	16	20	24	32	40	48	60	72
HC036001	0.74	0.70	0.58	0.39	0.67	1.09	0.48	0.63	0.64	0.30	0.62	0.38	0.22	0.61
HC036002	0.87	0.94	0.91	1.25	0.96	1.09	0.92	1.18	0.96	0.68	0.88	0.67	0.36	1.34
HC036003	0.68		0.53	0.49	0.76	0.80	0.75	1.01	0.97	0.56	0.57	0.64	0.70	1.05
HC036004	1.59	1.11	0.79	1.12	0.80	1.40	1.27	0.87	1.05	0.95	1.10	1.16	1.27	1.06
HC036005	1.38	1.29	1.29	1.67	1.36	1.09	1.30	1.31	1.18	1.55	1.28	1.45	1.50	1.73
HC036006	1.33	1.63	0.96	1.26	0.99	1.16	0.98	0.95	1.02	1.39	1.32	0.96	1.61	0.97
HC036007	1.12	1.19	1.19	0.82	0.92	0.58	0.97	1.29	0.88	1.32	1.24	0.96	0.92	1.01

**Table 4:** Log_10_ HTLV-1 proviral DNA (CSF): −2 is baseline evaluation and week 12 CSF was collected while on CsA treatment.

	Time since entry (weeks)	
	−2	12
HC036001	1.70	1.66
HC036002	1.51	0.87
HC036003	1.91	1.02
HC036004	1.62	1.34
HC036005	2.57	1.70
HC036006	1.63	1.60
HC036007	1.70	1.76

**Figure 8:**
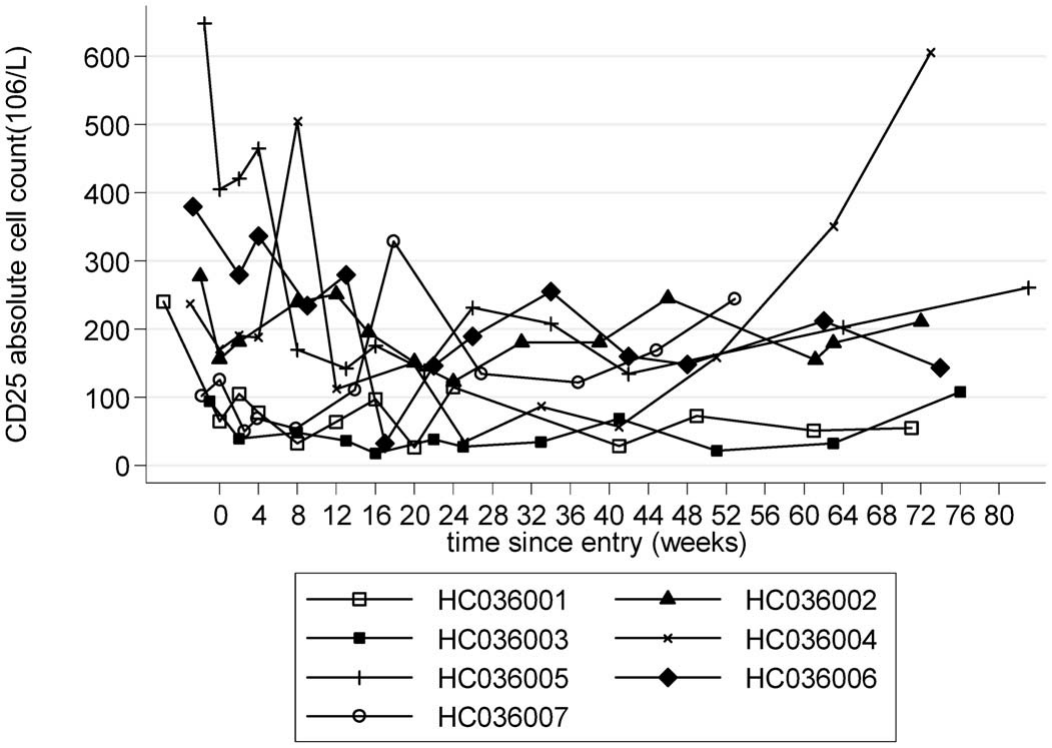
T cell activation marker: absolute CD4^+^/CD25^+^ T cell count (0–72 weeks).

The absolute counts of lymphocytes, CD4^+^ T-lymphocytes and CD8+ T lymphocytes gradually and consistently decreased during treatment with CsA particularly the CD4^+^ lymphocytes which were almost 300 cells/µL fewer on average at week 48 compared with baseline (p = 0.02, Table 2). These cell counts were still lower than baseline at week 72, i.e. 24 weeks after treatment cessation. The proportion of CD4^+^ lymphocytes expressing CD25^+^ also decreased, particularly during the first 12 weeks of therapy (mean (SE) decrease of −3.3% (2.6), p = 0.25, compared to baseline), remained lower than baseline through to 48 weeks and had returned towards baseline 24 weeks after CsA was stopped (Table 2). Changes were particularly marked in those patients who had an elevated frequency at baseline (Figure 8).

**Figure 9:**
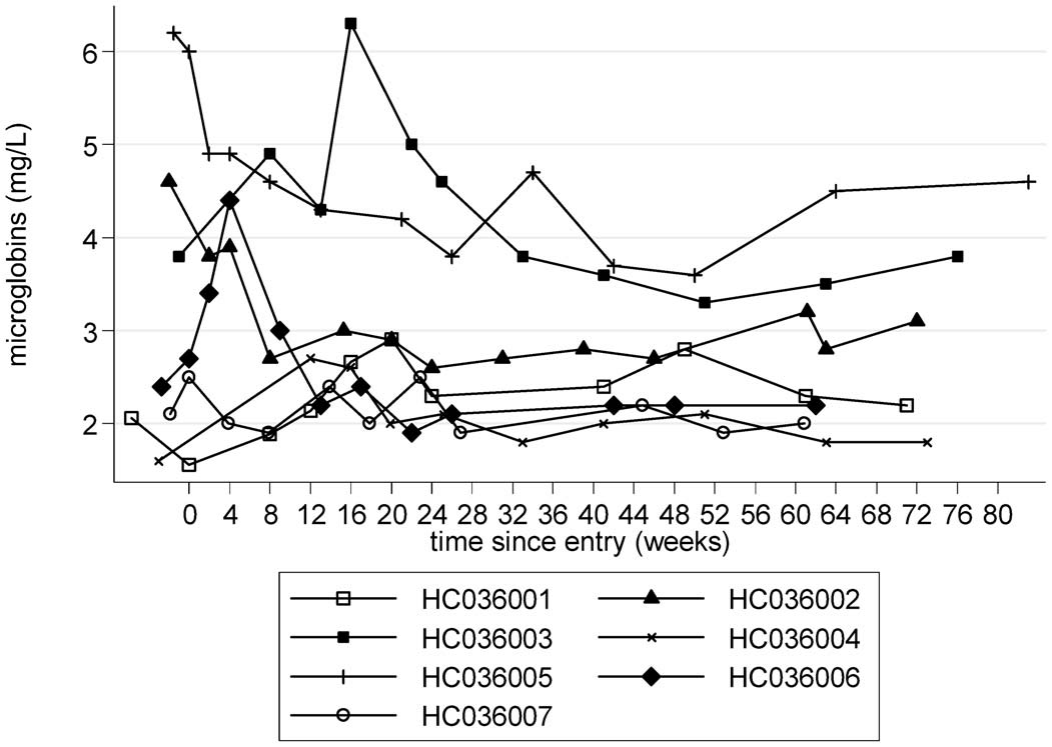
β_2_ microglobulin a marker of inflammation in peripheral venous blood (0–72 weeks).

Plasma β_2_ microglobulin, decreased with treatment and stayed at the 48 week level (mean (SE) decrease of −0.31 mg/L (0.39), p = 0.45, compared to baseline), without further treatment, at 72 weeks (Table 2). These changes were particularly marked in those patients who had elevated β_2_ microglobulin levels at baseline (Figure 9).

### Safety and tolerance

All patients remained normotensive with no evidence of nephrotoxicity.

Grade 1/2 serious adverse events were documented in four patients: one patient developed community acquired pneumonia at week 2, which was thought not to be due to CsA treatment, since she was known to have bronchiectasis and did not develop any further infections during 48 weeks of CsA treatment. One patient developed an acute urinary tract infection as well as acute depression during week 7. Both were not thought to be CsA related by the Data and Safety Monitoring Committee (DMSC), but the patient herself opted against CsA, which was stopped.

Tremor and headache, which were reported by the third patient at week 6, are well described side effects and their pathogenesis with CsA is not well understood. Tremor was the only side-effect that was thought to be truly due to CsA in this patient and it resolved with treatment cessation. The patient's headaches were adjudged to be possibly due to long term fungal sinus infection which was retrospectively reported on a pre-trial head CT scan. The headaches did not resolve when CsA was stopped.

### Concomitant medication taken during the trial

Although many concomitant medications were taken by the study participants both for symptomatic management of HAM/TSP and for co-existent pathologies, changes to treatments that could have influenced clinical outcomes, e.g. muscle relaxants for spasticity or sympathomimetics for urgency, were avoided during the study.

## Discussion

A recently published review of all therapy studies in patients with HAM/TSP found that only 86 patients with HAM/TSP have to date taken therapy within randomised controlled trials [11]. Two of these studies, enrolling a total of 40 patients, have found the approach of attempting to reduce HTLV-1 proviral burden by inhibiting HTLV-1 reverse transcriptase to improve symptoms, to be disappointing [23], [24]. Other open label, proof of concept studies with immune-modulators such as humanised anti-Tac (daclizumab) and interferon-β1 have reported a decrease in peripheral HTLV-1 DNA or tax RNA proviral load as well as some improvement of motor function with dacluzimab [25], [26].

Open, observational studies describe short term improvement in motor function of HAM/TSP patients with corticosteroids or interferon-α (IFNβ) [12], [13]. An open, randomised trial compared the efficacy of three dosages of IFNα and demonstrated transient, beneficial effects most marked with the highest dose [14].

The aim of the current study was to identify a safe, long-term, oral, corticosteroid sparing, anti-inflammatory and immunosuppressive treatment option for patients with HAM/TSP.

CsA, was selected to determine whether the course of a disease which is believed to be caused by heightened immune activation against chronic HTLV-1 infection could be modified by modulating T-cell activation, thereby giving further insight into pathogenesis.

The chief characteristic that distinguishes this trial from all other clinical trials in HAM/TSP are the very strict eligibility criteria, allowing recruitment of only those patients with early or progressing disease. It was considered that these patients would be most likely to benefit from medical intervention and that this would reduce the likelihood of under-estimating the potential of therapy. Although the comparison of the investigational drug with a placebo or another treatment option was not considered appropriate in the absence of any safety or efficacy data in patients with HAM/TSP, a comparison of on treatment data with each patient's his/her own repeat baseline measures are useful to identify a treatment signal in a small group of patients.

None of the patients were lost to follow up and all were followed up at the set time points prospectively aiming to capture pre, on and post treatment data. CsA's effect on disease was described by area under the curve analyses, a useful tool in summarising changes over time in a small number of patients whose measurements tend to fluctuate. However, due to the small sample size and number of outcomes analysed, the statistical results presented should be interpreted with caution.

The sample size is small for two reasons, one because of the stringent recruitment criteria, recruiting only “early and/or progressing” HAM/TSP and two to make sure this treatment, whilst known to be fairly safe and well tolerated given for other conditions, would also be safe in HTLV-1 infected patients who have a 4–6% background risk of developing ATLL.

This study focused on capturing specifically improvement in mobility as well as safety with CsA since these are the two most important outcomes to patients.

Reassuringly CsA was well tolerated by 5/7 patients for 48 weeks, did not have any severe adverse outcomes at 48 or 72 weeks and most importantly no patient has been diagnosed with any malignant disease or opportunistic infection during routine follow up to July 2011 (up to 5 years additional follow up).

**Figure 10:**
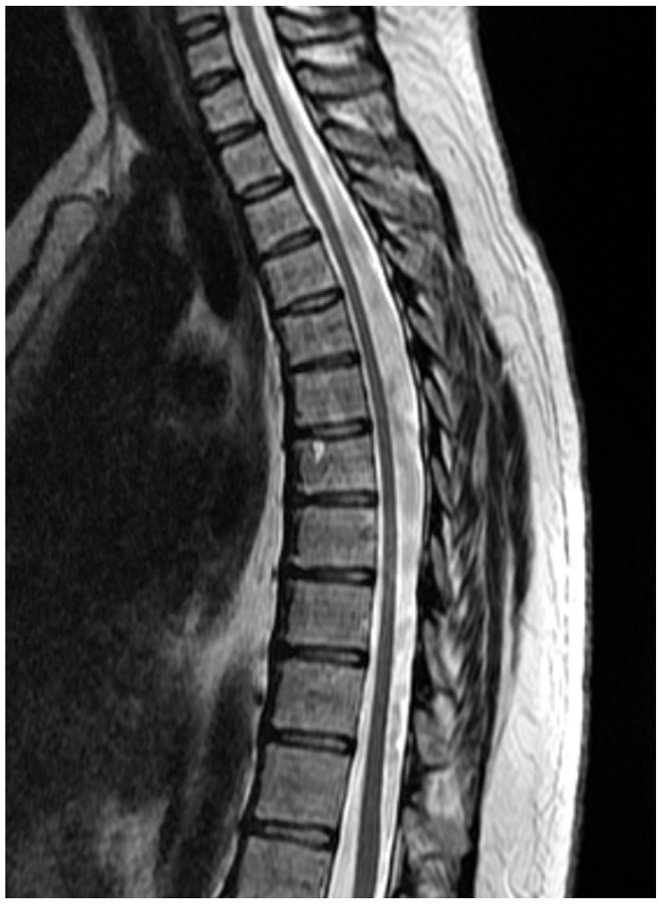
Magnetic resonance imaging of the thoracic chord (0–72 weeks). Clinical improvement was observed despite the marked atrophy of the thoracic cord.

We measured the time a patient takes to walk 10 meters, taking into account aid usage, as well as IPEC 1 disability scale as outcome measures apart from objective overall improvement. 10 m timed walk increased the sensitivity of measuring change in mobility, while IPEC 1, which like Osame's Motor Disability Scale (OMDS) was developed specifically for patients with HAM/TSP, included many other clinical symptoms of HAM/TSP, although it's assessment of gait is crude. In this study patients, whose mobility was deteriorating at baseline, showed an overall clinical improvement after initiating treatment with CsA within a short period of time (6 months). CsA treatment was associated with specific improvements in timed walk corrected for aid usage, pain, spasticity, daily urinary frequency and nocturia. This degree of improvement was unexpected, since five patients had been symptomatic for a long time (>2.5 to 17 years) including, as illustrated in Figure 10, patients with marked thoracic cord atrophy. Interestingly, despite the TDM driven dose adjustments CsA trough concentrations were below the target minimum on 33.9% of occasions, therefore the potential efficacy of CsA may have been underestimated in this study. Two patients opted to restart treatment during the follow up phase, due to worsening of symptoms after 48 weeks of treatment.

Although CsA treatment seemed to yield most clinical benefits by 24 weeks the laboratory markers continued to improve up to 48 weeks. In the subset of patients with high baseline concentrations plasma β_2_ microglobulin normalised. β_2_ microglobulin, which has recently been demonstrated to distinguish patients with HAM/TSP from HTLV-1 asymptomatic carriers with a relationship between concentration and degree of motor disability [27] should therefore be tested in larger clinical trials as a peripheral blood surrogate marker of HAM/TSP response to therapy. The observed reductions in β_2_ microglobulin and CD4^+^ CD25^+^ T cell frequency are encouraging both in their own right and particularly in association with the clinical findings. Furthermore the unexpected, albeit small reduction in HTLV-1 proviral load, which appears to be independent of the change in CD4^+^CD25^+^ frequency (data not shown) suggests that clinical benefits of immunosuppressive therapy were not at the expense of reduced HTLV-1 specific immunity. In theory a reduction HTLV-1 infected CD4^+^ CD25^+^ cells would be associated with a reduction in total HTLV-1DNA load but the reduction in CD4^+^ cells expressing CD25^+^ may also occur in HTLV-1 uninfected cells as a direct consequence of CsA therapy or even secondary to a reduced host response to secondary to a reduction in the number of cells capable of expressing viral proteins.

High HTLV-1 proviral load in CSF, compared with PBMCs, is recognised as a marker of HAM/TSP [28], [29]. The four-fold greater reduction in HTLV-1 proviral load in the CSF compared with PBMCs, therefore seems biologically significant, the more so for being independent of other, traditional markers of CSF inflammation such as lymphocyte counts and protein. One possibility is that with T-cell activation inhibition, fewer infected CD4^+^ T cells were migrating across the blood-brain barrier. This observation needs to be further tested in future studies including later sampling post therapy to determine the duration/reversibility of this change.

The optimal duration of therapy remains unknown. Whilst some participants maintained an improved clinical status after discontinuing therapy after 48 weeks others appeared to relapse early. Future, larger, clinical trials of CsA, should therefore consider treating patients for at least 48 weeks. One study participant recently relapsed again after stopping CsA for the second time, after 4 years of therapy.

In conclusion CsA, a steroid-sparing immunosuppressive agent, was associated with improved disease outcome in patients with early or progressing HAM/TSP at 24 and 48 weeks follow up. This finding suggests that activated lymphocytes contribute to pathogenesis. This study also demonstrates that close monitoring, even of patients with long standing HAM/TSP, is important to detect those with clinically progressive disease who may benefit from disease modifying therapy.

A multi-centre, international randomised controlled trial in patients with early and/or progressive HAM/TSP is the next step towards the establishment of an internationally acknowledged treatment protocol. Therefore the currently most commonly prescribed drug for active HAM/TSP, corticosteroids, as well as steroid sparing drugs such as INF-α, INF-β and CsA should be studied in larger phase 2 studies either in placebo-controlled or head-to-head comparative randomised controlled trials.

## Supporting Information

Appendix S1 **Modified Ashworth Scale Score (MASS).**
(DOCX)

Appendix S2 **Multiple sclerosis walking scale (MSWS-1).**
(DOCX)

Appendix S3 **Insituto de Pesquisa Clinica Evandro Chagas disability scores (IPEC).**
(DOCX)

Appendix S4 **Spasticity scale-88 score (SPAST-88).**
(DOCX)

Appendix S5 **Short form 36 health survey (SF-36).**
(DOCX)
