# Supplementary material for: Ciclosporin A Proof of Concept Study in Patients with Active, Progressive HTLV-1 Associated Myelopathy/Tropical Spastic Paraparesis
Source: PLoS Negl Trop Dis. 2012 Jun 12;6(6):e1675. doi: 10.1371/journal.pntd.0001675 (PMC3373656; doi:10.1371/journal.pntd.0001675)
Supplement: Appendix S1 — Modified Ashworth Scale Score (MASS). (DOCX) [file pntd.0001675.s001.docx]

# Appendix 1: Modified Ashworth Scale Score

0= No increase in muscle tone

1= Slight increase in muscle tone, manifested by a catch and release or by minimal resistance at the end of range of motion when the part is moved in flexion or extension /abduction or adduction, etc.

1+= Slight increase in muscle tone manifested by a catch, followed by minimal resistance throughout the remainder (Less than half) of the ROM

2= More marked increase in muscle tone through most of the ROM but the affected part is easily moved

3= Considerable increase in muscle tone, passive movement is difficult

4= Affected part is rigid in flexion or extension (abduction or adduction, etc)
