# Supplementary material for: Ciclosporin A Proof of Concept Study in Patients with Active, Progressive HTLV-1 Associated Myelopathy/Tropical Spastic Paraparesis
Source: PLoS Negl Trop Dis. 2012 Jun 12;6(6):e1675. doi: 10.1371/journal.pntd.0001675 (PMC3373656; doi:10.1371/journal.pntd.0001675)
Supplement: Appendix S2 — Multiple sclerosis walking scale (MSWS-1). (DOCX) [file pntd.0001675.s002.docx]

# Appendix 2: Multiple sclerosis walking scale (MSWS-1)

# *Date:………………*

- These questions ask about ***limitations to your walking during the past 2 weeks***.
- For each statement, please ***circle* the *one* number that best describes your degree of limitation**.
- Please answer ***all questions* even if some seem rather similar to others, or seem**

**irrelevant to you**.

- ***If you cannot walk at all*, please tick this box**.

| In the past 2 weeks how much has your condition | Not at all | A little | Moderately | Quite a bit | Extremely |
| --- | --- | --- | --- | --- | --- |
| 1. Limited your ability to walk? | 1 | 2 | 3 | 4 | 5 |
| 2. Limited your ability to run? | 1 | 2 | 3 | 4 | 5 |
| 3. Limited your ability to climb up and down the stairs? | 1 | 2 | 3 | 4 | 5 |
| 4. Made standing when doing things more difficult? | 1 | 2 | 3 | 4 | 5 |
| 5. Limited your balance when standing or walking? | 1 | 2 | 3 | 4 | 5 |
| 6. Limited how far you are able to walk? | 1 | 2 | 3 | 4 | 5 |
| 7. Increased the effort needed for you to walk? | 1 | 2 | 3 | 4 | 5 |
| 8. Made it necessary for you to use support when walking indoors (eg., holding on to furniture, using a stick, etc.)? | 1 | 2 | 3 | 4 | 5 |
| 9. Made it necessary for you to use support when walking outdoors (eg., using a stick a frame, etc.)? | 1 | 2 | 3 | 4 | 5 |
| 10. Slowed down your walking? | 1 | 2 | 3 | 4 | 5 |
| 11. Affected how smoothly you walk? | 1 | 2 | 3 | 4 | 5 |
| 12. Made you concentrate on your walking? | 1 | 2 | 3 | 4 | 5 |

**Total = / Average=**
