# Supplementary material for: Ciclosporin A Proof of Concept Study in Patients with Active, Progressive HTLV-1 Associated Myelopathy/Tropical Spastic Paraparesis
Source: PLoS Negl Trop Dis. 2012 Jun 12;6(6):e1675. doi: 10.1371/journal.pntd.0001675 (PMC3373656; doi:10.1371/journal.pntd.0001675)
Supplement: Appendix S3 — Insituto de Pesquisa Clinica Evandro Chagas disability scores (IPEC). (DOCX) [file pntd.0001675.s003.docx]

# Appendix 3: Insituto de Pesquisa Clinica Evandro Chagas disability scores (IPEC)

**1) THE IPEC DISABILITY SCALE (Physician’s reported)**

**MOTOR SCORE:**

Gait

0.Normal

1. Abnormal but can walk independently

2. Abnormal and dependent on eventual unilateral support

3. Abnormal and dependent on permanent unilateral support

4. Abnormal and dependent on eventual bilateral support

5. Abnormal and dependent on permanent bilateral support

6. Abnormal, dependent on permanent bilateral support, and occasional use of a wheelchair (WC)

7. Permanent use of a WC, stands up, and remains upright without support

8. Permanent use of a WC, uses arms to stand up, and remains upright without support

9. Permanent use of a WC, needs assistance from others to stand up and remain upright with support

10.Permanent use of a WC, unable to stand up, exhibits voluntary movements of the lower limbs

when seated

11.Permanent use of WC, unable to stand up, and does not have any voluntary movements of the

lower limbs

**Score: ______**

Running

0.Able to run

1.Unable to run

**Score: ______**

Climbing stairs

0.Climbs stairs

1.Climbs only when holding the handrail

2.Unable to climb

**Score: ______**

Jumping

0.Jumps on one or two feet

1.Jumps on two feet, but not with only one

2.Jumps on two feet only with hand support

3.Unable to jump

**Score: ______**

**SPASTICITY SCORE:**

Clonus

0.Absent

1.Only induced by the examiner

2.Spontaneous

**Score: ______**

Flexor / Extensor Spasms

0.Absent

1.Present

**Score: ______**

**SENSORY SCORE:**

Paresthesiae

0.Absent

1.Present, intermittently

2.Present, permanently

**Score: ______**

Lumbar and / or lower limb pain

0.Absent

1.Present, intermittently

2.Present during most of the day

**Score: ______**

**SPHINCTER SCORE:**

Bladder control

0.Total

1.Urgency, intermittently

2.Occasional incontinence or retention

3.Use of permanent catheter or regular use of relieve catheter

**Score: ______**

Bowel Continence

0.Normal

1.Constipation

2.Incontinence or total retention, needs manual extraction or enemas

**Score: ______**

**TOTAL SCORE: (0-29): ______**

**2) THE NEW IPEC DISABILITY SCALE**

***Patient’s self-assessment***

***All Questions Refer To The Last 2 Weeks***

**LOCOMOTION SCORE:**

| **Classify the degree of difficulty**  **that you have had for:** | | | **None** | | **Minimal** | | **Small** | | **Moderate** | | **A lot** | | **Extreme** | | **Unable** | |
| --- | --- | --- | --- | --- | --- | --- | --- | --- | --- | --- | --- | --- | --- | --- | --- | --- |
| **1.** | **Raising from a chair** | | 0 | | 1 | | 2 | | 3 | | 4 | | 5 | | 6 | |
| **2.** | **Remaining in the upright position for at least 5 minutes** | | 0 | | 1 | | 2 | | 3 | | 4 | | 5 | | 6 | |
| **3.** | **Moving around inside your house** | | 0 | | 1 | | 2 | | 3 | | 4 | | 5 | | 6 | |
| **4.** | **Moving around outside your house** | | 0 | | 1 | | 2 | | 3 | | 4 | | 5 | | 6 | |
| **5.** | **Going up and down stairs** | | 0 | | 1 | | 2 | | 3 | | 4 | | 5 | | 6 | |

| **How frequently do you need assistance (persons, sticks, wheelchair, etc) to do most of the activities above** | **Never** | | **Rarely** | | **Eventually** | | **Frequently** | | **Often** | | **Always** | |  |
| --- | --- | --- | --- | --- | --- | --- | --- | --- | --- | --- | --- | --- | --- |
| **+ (*)** | | 0 | | 0.25 | | 0.50 | | 0.75 | | 1 | | 1.25 | |

**SPHINCTER CONTROL:**

| **Classify the degree of difficulty**  **that you have had for:** | **None** | **Minimal** | **Small** | **Moderate** | **A lot** | **Extreme** | **Unable** |
| --- | --- | --- | --- | --- | --- | --- | --- |

| **1.** | **Controlling your bladder?** | 0 | 1 | 2 | 3 | 4 | 5 | 6 |
| --- | --- | --- | --- | --- | --- | --- | --- | --- |
| **2.** | **Defecating?** | 0 | 1 | 2 | 3 | 4 | 5 | 6 |

**Add Pain Score from the Routine Follow Up Sheet on a scale 0-6.**

**TOTAL:**

(*)= Add to the final scorE
