# Supplementary material for: Ciclosporin A Proof of Concept Study in Patients with Active, Progressive HTLV-1 Associated Myelopathy/Tropical Spastic Paraparesis
Source: PLoS Negl Trop Dis. 2012 Jun 12;6(6):e1675. doi: 10.1371/journal.pntd.0001675 (PMC3373656; doi:10.1371/journal.pntd.0001675)
Supplement: Appendix S4 — Spasticity scale-88 score (SPAST-88). (DOCX) [file pntd.0001675.s004.docx]

Appendix 4: Spasticity scale-88 score

| **SPASTICITY SCALE (SPAST-88)**  **[generic version of the Multiple Sclerosis Spasticity Scale (MSSS-88)]** |
| --- |

- This questionnaire asks how **bothered** you have been by your spasticity **in the past two weeks**.
- **By spasticity we mean muscle stiffness and spasms.**
- By **bothered** we mean how distressed or upset you have been by any of the following

problems.

- For each statement, please **circle** the **one** number that best describes how you feel.
- Please answer **all** questions even if some seem rather similar to others, or irrelevant to you.

**Section 1:**

**This section concerns muscle stiffness.**

| **As a result of your *spasticity*, how much in the past two weeks have you been bothered by:** | Not at all bothered | A little bothered | Moderately bothered | Extremely bothered |
| --- | --- | --- | --- | --- |
| **01. Stiffness when walking?** | 1 | 2 | 3 | 4 |
| **02. Stiffness anywhere in your lower limbs?** | 1 | 2 | 3 | 4 |
| **03. Stiffness when you are in the same position for a long time?** | 1 | 2 | 3 | 4 |
| **04. Stiffness first thing in the morning?** | 1 | 2 | 3 | 4 |
| **05. Tightness anywhere in your lower limbs?** | 1 | 2 | 3 | 4 |
| **06. Your lower limbs feeling rigid?** | 1 | 2 | 3 | 4 |
| **07. Stiffness when standing up?** | 1 | 2 | 3 | 4 |
| **08. Tightness in your muscles?** | 1 | 2 | 3 | 4 |
| **09. Stiffness that is unpredictable?** | 1 | 2 | 3 | 4 |
| **10. Feeling that your muscles are pulling?** | 1 | 2 | 3 | 4 |
| **11. Stiffness in your whole body?** | 1 | 2 | 3 | 4 |
| **12. Your whole body feeling rigid?** | 1 | 2 | 3 | 4 |

**Section 2:**

**This section concerns pain and discomfort.**

| **As a result of your *spasticity*, how much in the past two weeks have you been bothered by:** | Not at all bothered | A little bothered | Moderately bothered | Extremely bothered |
| --- | --- | --- | --- | --- |
| **13. Feeling restricted and uncomfortable?** | 1 | 2 | 3 | 4 |
| **14. Feeling uncomfortable sitting for a long time?** | 1 | 2 | 3 | 4 |
| **15. Painful or uncomfortable spasms?** | 1 | 2 | 3 | 4 |
| **16. Pain when in the same position for too long?** | 1 | 2 | 3 | 4 |
| **17. Feeling uncomfortable lying down for a long time?** | 1 | 2 | 3 | 4 |
| **18. Difficulties finding a comfortable position to sleep in bed?** | 1 | 2 | 3 | 4 |
| **19. Pain in the muscles on getting out of bed in the morning?** | 1 | 2 | 3 | 4 |
| **20. Pain in the muscles provoked by movement?** | 1 | 2 | 3 | 4 |
| **21. Constant pain in the muscles?** | 1 | 2 | 3 | 4 |

**Section 3:**

**This section concerns muscle spasms.**

| **As a result of your *spasticity*, how much in the past two weeks have you been bothered by:** | Not at all bothered | A little bothered | Moderately bothered | Extremely bothered |
| --- | --- | --- | --- | --- |
| **22. Spasms that come on unpredictably?** | 1 | 2 | 3 | 4 |
| **23. Powerful or strong spasms?** | 1 | 2 | 3 | 4 |
| **24. Spasms when first getting out of bed in the morning?** | 1 | 2 | 3 | 4 |
| **25. Spasms provoked by changing positions?** | 1 | 2 | 3 | 4 |
| **26. Spasms provoked by movement?** | 1 | 2 | 3 | 4 |
| **27. Spasms where your leg kicks out in front of you?** | 1 | 2 | 3 | 4 |
| **28. Spasms provoked by certain positions?** | 1 | 2 | 3 | 4 |
| **29. Spasms disturbing sleep?** | 1 | 2 | 3 | 4 |
| **30. Spasms when doing certain tasks?** | 1 | 2 | 3 | 4 |
| **31. Spasms when travelling over bumps or cobbles?** | 1 | 2 | 3 | 4 |
| **32. Spasms where your knees pull up?** | 1 | 2 | 3 | 4 |
| **33. Spasms causing legs to hit things?** | 1 | 2 | 3 | 4 |
| **34. Spasms provoked by touch?** | 1 | 2 | 3 | 4 |
| **35. Spasms pushing you out of a chair or wheelchair?** | 1 | 2 | 3 | 4 |

**Section 4:**

**This section concerns the effect of spasticity on your daily activities.**

| **As a result of your spasticity, how much have you been limited in your ability over the past two weeks to carry out the following daily activities?** | Not at all limited | A little limited | Moderately limited | Extremely limited |
| --- | --- | --- | --- | --- |
| **36. Putting on your socks or shoes?** | 1 | 2 | 3 | 4 |
| **37. Doing housework such as cooking or cleaning?** | 1 | 2 | 3 | 4 |
| **38. Getting in and out of a car?** | 1 | 2 | 3 | 4 |
| **39. Getting in and out of shower and/or bath?** | 1 | 2 | 3 | 4 |
| **40. Sitting up in bed?** | 1 | 2 | 3 | 4 |
| **41. Getting into or out of bed?** | 1 | 2 | 3 | 4 |
| **42. Turning over in bed?** | 1 | 2 | 3 | 4 |
| **43. Getting into or out of a chair?** | 1 | 2 | 3 | 4 |
| **44. Getting dressed or undressed?** | 1 | 2 | 3 | 4 |
| **45. Getting on or off the toilet seat?** | 1 | 2 | 3 | 4 |
| **46. Drying yourself with a towel?** | 1 | 2 | 3 | 4 |

**Section 5:**

**^[[1]](#footnote-1)^**

**This section concerns the effect of spasticity on your ability to walk.**

| **If you cannot take any steps at all, even with help,**  **please tick this box and ignore questions 47 to 56.** |  |
| --- | --- |

| **As a result of your spasticity, how much in the past two weeks have you been bothered by:** | Not at all bothered | A little bothered | Moderately bothered | Extremely bothered |
| --- | --- | --- | --- | --- |
| **47. Difficulties walking smoothly?** | 1 | 2 | 3 | 4 |
| **48. Being slow when walking?** | 1 | 2 | 3 | 4 |
| **49. Having to concentrate on your walking?** | 1 | 2 | 3 | 4 |
| **50. Having to increase the effort needed for you to walk?** | 1 | 2 | 3 | 4 |
| **51. Being slow when going up or down stairs?** | 1 | 2 | 3 | 4 |
| **52. Being clumsy when walking?** | 1 | 2 | 3 | 4 |
| **53. Tripping over or stumbling when walking?** | 1 | 2 | 3 | 4 |
| **54. Feeling like you are walking through treacle?** | 1 | 2 | 3 | 4 |
| **55. Losing your confidence to walk?** | 1 | 2 | 3 | 4 |
| **56. Feeling embarrassed to walk?** | 1 | 2 | 3 | 4 |

SPASTICITY SCALE (SPAST-88) © 2006 Peninsula Medical School, Devon, UK

**Section 6:**

**This section concerns the effect of spasticity on your body movement.**

| **As a result of your spasticity, how much in the past two weeks have you been bothered by:** | Not at all bothered | A little bothered | Moderately bothered | Extremely bothered |
| --- | --- | --- | --- | --- |
| **57. Difficulties moving freely?** | 1 | 2 | 3 | 4 |
| **58. Difficulties moving smoothly?** | 1 | 2 | 3 | 4 |
| **59. Limited range of movement?** | 1 | 2 | 3 | 4 |
| **60. Difficulties moving parts of your body?** | 1 | 2 | 3 | 4 |
| **61. Difficulties bending your limbs?** | 1 | 2 | 3 | 4 |
| **62. Your body being resistant to movement?** | 1 | 2 | 3 | 4 |
| **63. Your body or limbs feeling locked?** | 1 | 2 | 3 | 4 |
| **64. Awkward or jerky movement?** | 1 | 2 | 3 | 4 |
| **65. Difficulties straightening your limbs?** | 1 | 2 | 3 | 4 |
| **66. Difficulties relaxing parts of your body?** | 1 | 2 | 3 | 4 |
| **67. No control over your body?** | 1 | 2 | 3 | 4 |

**Section 7:**

**This section concerns the effect of spasticity on your feelings.**

| **As a result of your spasticity, how much in the past two weeks have you been bothered by:** | Not at all bothered | A little bothered | Moderately bothered | Extremely bothered |
| --- | --- | --- | --- | --- |
| **68. Feeling frustrated?** | 1 | 2 | 3 | 4 |
| **69. Feeling less confident in yourself?** | 1 | 2 | 3 | 4 |
| **70. Feeling inadequate?** | 1 | 2 | 3 | 4 |
| **71. Feeling low?** | 1 | 2 | 3 | 4 |
| **72. Feeling irritated?** | 1 | 2 | 3 | 4 |
| **73. Feeling angry?** | 1 | 2 | 3 | 4 |
| **74. Feeling depressed?** | 1 | 2 | 3 | 4 |
| **75. Loss of self-worth?** | 1 | 2 | 3 | 4 |
| **76. Feeling like a failure?** | 1 | 2 | 3 | 4 |
| **77. Feeling frightened?** | 1 | 2 | 3 | 4 |
| **78. Crying (tearful)?** | 1 | 2 | 3 | 4 |
| **79. Feeling panicky?** | 1 | 2 | 3 | 4 |
| **80. Feeling nervous?** | 1 | 2 | 3 | 4 |

SPASTICITY SCALE (SPAST-88) © 2006 Peninsula Medical School, Devon, UK

**Section 8:**

**This section concerns the effect of spasticity on your social functioning.**

| **As a result of your spasticity, how much in the past two weeks have you been bothered by:** | Not at all bothered | A little bothered | Moderately  bothered | Extremely bothered |
| --- | --- | --- | --- | --- |
| **81. Difficulties going out?** | 1 | 2 | 3 | 4 |
| **82. Feeling isolated?** | 1 | 2 | 3 | 4 |
| **83. Feeling vulnerable?** | 1 | 2 | 3 | 4 |
| **84. Difficulties finding energy for other people?** | 1 | 2 | 3 | 4 |
| **85. Feeling reluctant to go out?** | 1 | 2 | 3 | 4 |
| **86. Feeling less sociable?** | 1 | 2 | 3 | 4 |
| **87. Difficulties with relationships with other family members?** | 1 | 2 | 3 | 4 |
| **88. Difficulties interacting with people?** | 1 | 2 | 3 | 4 |

^[[2]](#footnote-2)^

1. SPASTICITY SCALE (SPAST-88) © 2006 Peninsula Medical School, Devon, UK [↑](#footnote-ref-1)
2. SPASTICITY SCALE (SPAST-88) © 2006 Peninsula Medical School, Devon, UK [↑](#footnote-ref-2)
