# Supplementary material for: Ciclosporin A Proof of Concept Study in Patients with Active, Progressive HTLV-1 Associated Myelopathy/Tropical Spastic Paraparesis
Source: PLoS Negl Trop Dis. 2012 Jun 12;6(6):e1675. doi: 10.1371/journal.pntd.0001675 (PMC3373656; doi:10.1371/journal.pntd.0001675)
Supplement: Appendix S5 — Short form 36 health survey (SF-36). (DOCX) [file pntd.0001675.s005.docx]

**Appendix 5: Short form 36 health survey (SF-36)**

1. **IN GENERAL WOULD YOU SAY YOUR HEALTH IS: (Please tick one)**

EXCELLENT VERY GOOD GOOD FAIR POOR

1. **COMPARED TO ONE YEAR AGO, HOW WOULD YOU RATE YOUR HEALTH IN GENERAL?**

**(Please tick one)**

MUCH BETTER THAN ONE YEAR AGO

SOMEWHAT BETTER THAN ONE YEAR AGO

ABOUT THE SAME

SOMEWHAT WORSE

MUCH WORSE THAN ONE YEAR AGO

| **THE FOLLOWING QUESTIONS AE ABOUT ACTIVITIES YOU MIGHT DO DURING A TYPICAL DAY.**  **DOES YOUR HEALTH LIMIT YOU A LOT IN THESE ACTIVITIES? IF SO, HOW MUCH?** |
| --- |

**(Please tick one box on each line)**  **YES YES NO, NOT**

**LIMITED LIMITED LIMITED**

**A LOT A LITTLE AT ALL**

1. **VIGOROUS ACTIVITIES**

**(Such as running, lifting heavy objects, or**

**participating in strenuous sports)**

1. **MODERATE ACTIVITIES**

**(Such as moving a table, pushing a vacuum**

**cleaner, bowling or playing golf)**

1. **LIFTING OR CARRYING GROCERIES**
2. **CLIMBING SEVERAL FLIGHTS OF STAIRS**
3. **CLIMBING ONE FLIGHT OF STAIRS**
4. **BENDING, KNEELING OR STOOPING**
5. **WALKING MORE THAN A MILE**
6. **WALKING HALF A MILE**
7. **WALKING 100 YARDS**
8. **BATHING OR DRESSING YOURSELF**

| **DURING THE PAST 4 WEEKS HAVE YOU HAD ANY OF THE FOLLOWING PROBLEMS WITH YOUR**  **WORK OR OTHER DAILY REGULAR ACTIVITIES AS A RESULT OF YOUR PHYSICAL HEALTH?** |
| --- |

**(Please answer YES or NO for each problem) YES NO**

1. **CUT DOWN ON THE AMOUNT OF TIME YO SPENT**

**ON WORK OR OTHER ACTIVITIES**

1. **ACCOMPLISHED LESS THAN YOU WOULD LIKE**
2. **WERE LIMITED IN THE KIND OF WORK OR OTHER ACTIVITIES**
3. **HAD DIFIICULTY PERFORMING THE WORK OR OTHER**

**ACTIVITIES? (e.g. it took extra effort)**

| **DURING THE PAST 4 WEEKS HAVE YOU HAD ANY OF THE FOLLOWING PROBLEMS WITH YOUR WORK OR OTHER DAILY REGULAR ACTIVITIES AS A RESULT OF YOUR EMOTIONAL HEALTH (SUCH AS FEELING DEPRESSED OR ANXIOUS)?** |
| --- |

**YES NO**

**17. CUT DOWN ON THE AMOUNT OF TIME YOU SPENT ON WORK**

**OR ACTIVITIES?**

**18. ACCOMPLISHIED LESS THAN YOU WOULD LIKE?**

1. **DIDN’T DO WORK OR OTHER ACTIVITIES AS CAREFULLY AS**

**USUAL?**

1. **DURING THE PAST 4 WEEKS TO WHAT EXTENT HAS YOUR PHYSICAL HEALTH OR EMOTIONAL PROBLEMS INTERFERED WITH FAMILY, FRIENDS, NEIGHBOURS OR GROUPS? (please tick one)**

| NOT AT ALL | SLIGHTLY | MODERATELY | QUITE A BIT | EXTREMELY |
| --- | --- | --- | --- | --- |
|  |  |  |  |  |

**21. HOW MUCH BODILY PAIN HAVE YOU HAD IN THE PAST 4 WEEKS?**

**(please tick one)**

| NONE | VERY MILD | MILD | MODERATE | SEVERE | VERY SEVERE |
| --- | --- | --- | --- | --- | --- |
|  |  |  |  |  |  |

**22. DURING THE PAST 4 WEEKS HOW MUCH DID PAIN INTERFERE WITH YOUR NORMAL WORK OR HOUSEWORK?**

**(Please tick one)**

| NOT AT ALL | A LITTLE BIT | MODERATELY | QUITE A BIT | EXTREMELY |
| --- | --- | --- | --- | --- |
|  |  |  |  |  |

| **These questions are about how you feel and how things have been with you during the past month.**  **(For each question, please indicate the one answer that comes closest to the way you have been feeling)** |
| --- |

**(Please tick one box on each line)**

| **How much time during the past month:** | **All of the time** | **Most of the time** | **A good bit of**  **the time** | **Some of the time** | **A little of the time** | **None of the time** |
| --- | --- | --- | --- | --- | --- | --- |

| **23.** | **Did you feel full of life?** |  |  |  |  |  |  |
| --- | --- | --- | --- | --- | --- | --- | --- |
| **24.** | **Have you been a very nervous person?** |  |  |  |  |  |  |
| **25.** | **Have you felt so down in the dumps that nothing could cheer you up?** |  |  |  |  |  |  |
| **26.** | **Have you felt calm and peaceful?** |  |  |  |  |  |  |
| **27.** | **Did you have a lot of energy?** |  |  |  |  |  |  |
| **28.** | **Have you felt downhearted and low?** |  |  |  |  |  |  |
| **29.** | **Did you feel worn out?** |  |  |  |  |  |  |
| **30.** | **Have you been a happy person?** |  |  |  |  |  |  |
| **31.** | **Did you feel tired?** |  |  |  |  |  |  |
| **32.** | **Has your health limited your social activities (Like visiting close friends or close relatives)?** |  |  |  |  |  |  |

| **Please choose the answer that best describes how true or false each of the following statements is for you.** |
| --- |

**(Please tick one box on each line)**

|  | **Definitely**  **true** | **Mostly**  **true** | **Not**  **sure** | **Mostly**  **false** | **Definitely**  **false** |
| --- | --- | --- | --- | --- | --- |

| **33.** | **I seem to get ill more easily than other people** |  |  |  |  |  |
| --- | --- | --- | --- | --- | --- | --- |
| **34.** | **I am as healthy as anybody I know** |  |  |  |  |  |
| **35.** | **I expect my health to get worse** |  |  |  |  |  |
| **36.** | **My health is excellent** |  |  |  |  |  |

Reproduced by kind permission of the Health Outcomes Institute, Minneapolis, USA.
